# The Impact of Harsh Parenting on the Development of Obesity in Adulthood: An Examination of Epigenetic/Gene Expression Mediators Among African American Youth

**DOI:** 10.3389/fcvm.2021.755458

**Published:** 2021-11-05

**Authors:** Man-Kit Lei, Steven R. H. Beach, Ronald L. Simons, Kaixiong Ye

**Affiliations:** ^1^Department of Sociology, University of Georgia, Athens, GA, United States; ^2^Department of Psychology, Center for Family Research, University of Georgia, Athens, GA, United States; ^3^Department of Sociology, University of Georgia, Athens, GA, United States; ^4^Department of Genetics, University of Georgia, Athens, GA, United States

**Keywords:** harsh parenting, body mass index, risky family model, obesity-related DNA methylation, gene expression of obesity, mediating pathway

## Abstract

**Objective:** We examined the association of prospectively assessed harsh parenting during adolescence with body mass index (BMI) in young adulthood among African American youth. We also assessed the role of methylation of obesity-related genes and gene expression markers of obesity as mediators of this association, providing a pathway for the biological embedding of early harsh parenting and its long-term impact on young adult health.

**Methods:** Hypotheses were tested with a sample of 362 African American youth for whom harsh parenting was assessed at ages 10–15, BMI was assessed at age 10 and 29, and both DNA methylation (DNAm) and gene expression of obesity genes were assessed at age 29. Mediational analyses were conducted using bootstrap methods to generate confidence intervals.

**Results:** Controlling for genetic risk for obesity and health-related covariates, harsh parenting across childhood and adolescence was associated with change in BMI (Δ BMI) from ages 10–29. In addition, we found that the indirect effect of harsh parenting on Δ BMI was mediated through obesity-related DNAm and accounted for 45.3% of the total effect. Further, obesity-related DNAm mediated the effect of harsh parenting on gene expression of obesity-related genes (GEOG), and GEOG, in turn, mediated the impact of obesity-related DNAm on ΔBMI. This pathway accounted for 3.4% of the total effect. There were no gender differences in the magnitude of this indirect effect.

**Conclusions:** The results suggest that alterations in methylation and gene expression mediate the impact of harsh parenting on change in obesity from childhood to young adulthood, illustrating plausible biological pathways from harsh parenting to obesity and bolstering the hypothesis that harsh parenting in childhood and adolescence can become biologically embedded and contribute to obesity.

## Introduction

The prevalence of obesity has increased dramatically during several decades in the United States and other developed countries (1). This concerning development has particular relevance for the health of Black Americans, who experience a greater prevalence of obesity relative to non-Hispanic Whites at every age (2). Given that obesity confers increased health risks across many chronic diseases of aging (3–5), especially cardiovascular diseases (CVD) (6–8), body mass index (BMI), a widely used indicator of obesity, is a key outcome. BMI is a predictor of cardiometabolic risk and has strong associations with both morbidity and mortality (9). Prospective studies suggest that every 5 kg/m^2^ increase in BMI is associated with a 30% increase in the risk of all-cause mortality (10). Obesity is, therefore, a major public health threat and a contributor to health disparities.

Recent studies suggest that early family environments, especially those that are stressful for youth, may play a significant role in the development of obesity in adulthood (6, 11–13). The risky family model provides a theoretical account of the way that early family stress carries forward over the life course to have an impact on health (14). This model assumes that some family environments put youth at risk for later health problems by creating emotionally unsupportive rearing climates, this is thought to result from harsh parenting practices (15). Such harsh family environments are hypothesized to trigger the development of the physiological stress response systems, calibrating how individuals respond to threats throughout the life span (16). When these stresses evoke perceptions of threat, they have the potential to activate the hypothalamic-pituitary-adrenal (HPA) axis, causing alterations in a range of tissues. Chronic and recurrent activation of these changes can have long-term consequences for adult health outcomes (11) *via* multiple mechanisms, including alterations in fat deposition contributing to increased weight gain. Currently unknown is the extent to which sources of family stress may exert their effect by influencing methylation and gene expression mechanisms. If so, identifying mediators of the impact of harsh parenting during adolescence on adult BMI has the potential to strengthen and expand current models of social adversity on obesity, and identify new points of intervention.

Deoxyribonucleic acid methylation (DNAm), one of the major epigenetic mechanisms, may provide a biological pathway connecting parenting practices with obesity and well-being, and potentially account for long-lasting effects. DNAm occurs when “a methyl group attaches to a segment of DNA at a CpG site (i.e., a DNA region where a cytosine nucleotide is positioned next to a guanine nucleotide separated by one phosphate)” (17, 18). In recent years there has been significant progress in the use of DNAm to identify markers of health and aging (19). Yet, only recently have researchers have identified obesity-related methylation changes among African Americans. Wang et al. (20) identified five CpG sites (cg18181703, cg09349128, cg06178669, cg21585138, and cg03257930) associated with obesity among African Americans that survived extensive controls and were also associated with gene expression. These five CpG sites can be used to create a DNAm index of obesity that should also predict gene expression associated with obesity.

Although broad patterns of DNAm are established during early embryonic and fetal life, several lines of research demonstrate that methylation also can be influenced by environmental factors throughout the life span (21), perhaps especially during some periods of rapid developmental change, such as adolescence. Thus, it is possible for adverse experiences in adolescence to influence methylation of obesity-related genes (turned them on *via* hypomethylation or off *via* hypermethylation). Building upon research showing that harsh parenting can impact epigenetic effects (22), we hypothesized that exposure to harsh parenting in adolescence would foster obesity-related DNAm. This change in methylation, in turn, was expected to be related to obesity in adulthood.

Further, based on the prior findings of Wang et al. (20), individual differences in obesity-related DNAm were expected to predict individual differences in gene expression (23) for the five genes represented on the index. Given that DNAm can be influenced by social environments and influence “the genome to express (either up-regulate or down-regulate) particular genes” (21, 24), several lines of research converge to support the hypothesis that changes in gene expression related to changes in DNAm may be influenced by family environment (20, 25–27). However, we are aware of only one study that has investigated the association between DNA methylation, gene expression, and obesity. Using an African American sample, Wang et al. (20) provided evidence that the gene expression levels of five genes (SOCS3, CISH, PIM3, KLF4, and HRASLS2) were significantly associated with BMI and obesity-related DNAm. Accordingly, one way in which harsh parenting may influence obesity in adulthood is by acting on gene expression *via* DNA methylation change.

**Figure 1:**
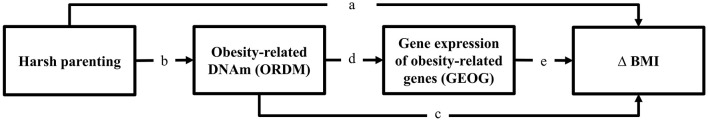
Theoretical model showing hypothesized indirect pathways from harsh parenting in adolescence to change in BMI through obesity-related DNAm and gene expression of obesity-related genes. Δ = change in BMI from ages 10 (wave 1) to 29 (wave 7).

Using a multiple wave prospective research design that spanned 20 years, we tested the general model shown in Figure 1. Consistent with the risky family model, we first hypothesized that harsh parenting in adolescence would forecast significantly greater increases in BMI from ages 10–29 (Δ BMI) (Pathway a). Then, we expected that much of the effect of harsh parenting on BMI would be mediated DNAm-based obesity (ORDM) index. Finally (Pathway b and c), we added gene expression of obesity-related genes (GEOG) as an additional mediator and hypothesized that the ORDM would mediate the effect of harsh parenting on GEOG, and GEOG, in turn, would mediate the link between ORDM and Δ BMI (Pathway b, d, and e).

## Materials and Methods

### Subjects

We tested the hypotheses using data from the seven waves of the Family and Community Health Study (FACHS). At the first wave (1997–1998), the FACHS sample consisted of 889 African American fifth-grade children (28). Their mean age was 10.56 years (*SD* = 0.631; range 9–13). The second, third, fourth, fifth, and sixth waves of data were collected in 1999–2000, 2002–2004, 2005–2007, 2008–2009, and 2010–2011, capturing information when youth were mean ages 12.5, 15.7, 18.8, 21.5, and 23.5, respectively. In 2015–2016 the 7th Wave of data collection was completed that included blood draws. The mean ages were about 29 years (*SD* = 0.803; range 28–32). Given the logistics of scheduling home visits by phlebotomists, only members of the sample residing in Georgia, Iowa, or a contiguous state were identified as eligible. After also excluding persons who were deceased, incarcerated, or otherwise unreachable, we were left with a pool of 556 individuals, 470 (86%) of whom agreed to provide blood. In the current study, analyses are based on the 362 respondents (131 men and 231 women) who reported BMI at age 29 and who were successfully assayed for genotypes, methylation, and gene expression. Comparisons of this subsample (*n* = 362) with those who were not included in the analysis (*n* = 527) did not reveal any significant differences with regard to harsh parenting, BMI, family income, and parental education at wave 1 (see online Supplementary Table 1). The complete data set for the current study can be downloaded from Supplementary Materials.

### Procedures

The protocol and all study procedures were approved by the University of Georgia Institutional Review Board (Title: FACHS weathering-Targets, Study approval number 00006152). Computer-assisted interviews were administered in the respondent's home and took on average about 2 h to complete. The instruments were presented on laptop computers. Questions appeared in sequence on the screen, which both the researcher and participant could see. The researcher read each question aloud, and the participant entered an anonymous response using a separate keypad. Participants were also asked to provide a blood sample at age 29. The phlebotomist drew four tubes of blood (30 mL) from each participant; these were shipped on the same day to a laboratory for preparation. Whole blood DNA was prepared using cold protein precipitation, quantified with a NanoDrop photometer (Thermofisher, 168 Third Avenue Waltham, MA, USA), and stored at −20°C until use (Lahiri & Nurnberger Jr, 1991). To obtain mRNA values, blood samples were collected in a PAXgene tube and frozen and stored at −80°C until use.

### Measures

#### Harsh Parenting

At waves 1–3, respondents answered 14 questions regarding how often during the preceding year the primary caregiver engaged in behaviors such as shouting, criticizing, lecturing, and physical aggression (29). Response categories ranged from 1 = never to 4 = always. Harsh parenting was coded so that higher scores indicated greater hostility and aggression. Coefficient alpha was 0.690 at wave 1, 0.780 at wave 2, and 0.814 at wave 3. Scores were standardized and then averaged across waves to form a composite measure of persistent exposure to harsh parenting.

#### Body Mass Index

At age 10 (wave 1), children's height and weight were reported to the interviewer. At age 29 (wave 4), the respondent's height and weight were measured at the time of the home visit. The Center for Disease Control calculator (https://www.cdc.gov/healthyweight/assessing/bmi/adult_bmi/english_bmi_calculator/bmi_calculator.html) was used to calculate BMI as weight in kilograms divided by the square of height in meters. We formulated a measure of change in BMI (Δ BMI) using the unstandardized residuals from the regression of BMI at wave 7 (age 10) on BMI at wave 1 (age 10).

#### Obesity-Related DNA Methylation

The Illumina Infinium HumanMethylationEPIC 850 BeadChip was used to assay genome-wide DNA methylation. This array contains 865,918 probes recognizing CpG positions of known transcripts, potential transcripts or CpG islands. Participants were randomly assigned to 16 sample “slides/chips” with groups of eight slides being bisulfite converted in a single plate, resulting in two “batches/plates.” A replicated sample of DNA was included in each plate to aid in assessment of batch variation and to ensure correct handling of specimens. The replicate sample was examined for average correlation of beta values between plates and was found to be >0.99. Quantile normalization methods were used, with separate normalization of Type I and Type II assays. This approach has been found to produce marked improvement for the Illumina array in detecting relationships by correcting distributional problems inherent in the manufacturer's default method for calculating the beta value. The beta value at each CpG locus was calculated as the ratio of the intensity of the methylated probe to the sum of intensities of the methylated and unmethylated probes. Finally, beta values after quantile normalization were used. We assessed DNAm-based obesity (ORDM) using a five CpG index (cg18181703, cg09349128, cg06178669, cg21585138, and cg03257930) developed by Wang et al. (20) for African Americans. The composite index of ORDM was calculated by averaging the beta values of the five CpGs. The mean was 0.366.

#### Gene Expression of Obesity-Related Genes

All available young adult PAXgene tube samples were sent to the Rutgers repository. The viable samples were processed using the Illumina HumanHT-12 v4 BeadChip. In each case, 200 ng of total RNA was processed according to the protocol supplied by Illumina. All samples were randomized prior to array hybridization using either two or three technical replicates. After background subtraction, raw Illumina probe data were exported using Illumina GenomeStudio v2011.1 software. The microarray data set of 47,323 probes was filtered by removing probes with detection threshold of *p* < 0.05, and probes with fewer than three beads present were also excluded, leaving 44,846 probes for analysis. Then, robust multiarray average normalized data were log2 transformed after quantile normalization and the quality of the microarray images was inspected visually using the ArrayAnalysis quality control pipeline (www.arrayanalysis.org). The results showed that there were no significant batch effects after quantile normalization.

GEOG was calculated for each participant based on the five transcripts of the obesity-related genes identified by Wang et al. (20): four genes (SOCS3, PIM3, CISH, and KLF4) were weighted +1 as positive indicators of BMI, and HRASLS2 was weighted −1 as an inverse indicator of obesity (20). The composite index of GEOG was calculated by averaging the log2 transformed values of the five transcripts of obesity-related genes.

#### Genetic Risk Score for Obesity Index

To examine and control the impact of background genetic variation on obesity, analyses were controlled for GRSO index developed by Monda et al. (30). The index of GRSO has been shown to associate with obesity, measured by BMI. In this study, blood was genotyped using the Illumina Infinium Multi- Ethnic Genotyping Array (MEGA). The eight SNPs included were located on eight different genes (rs543874 on SEC16B; rs6545800 on ADCY3; rs348495 on GNPDA2; rs7708584 on GALNT10; rs974417 on KLHL32; rs10261878 on MIR148A-NFE2L3; rs17817964 on FTO; and rs6567160 on MC4R). All SNPs were found to be in Hardy–Weinberg equilibrium. The weighted risk score weights the number of risk alleles present at each SNP (0, 1, 2) by its corresponding effect size estimated using previous study. Details regarding measuring of GRSO is described by Beach et al. (31).

#### Covariates

To account for variables that could provide plausible rival explanations, we controlled for *gender, income, unhealthy diet, exercise*, and *C-reactive protein (CRP)* at the last wave (age 29, wave 7). We also employed the last observation carried forward approach for imputing missing values at wave 7. *Income* was assessed by asking participants to report their income in the past year. Unhealthy diet was measured using a three-item scale that asked respondents to report how often in the past seven days (1 = none; 6 = more than once every day) they: (1) ate starchy foods like potatoes, peas, corn, rice, or noodles; (2) ate sweets such as candy bars, cake, cookies, or sugar-sweetened soda; and (3) ate fatty food like potato chips, corn chips, French fries, has browns, or Tater Tots. Scores on the three items were averaged to form the unhealthy diet measure. *Exercise* was measured with two items (e.g., on how many of the past 7 days did you exercise or participate in physical activity for at least 30 min that made you breathe hard such as running or riding a bicycle hard?) The response categories ranged from 1 (0 days) to 5 (all 7 days). Scores on the two items were averaged to form the exercise measure. C-reactive protein (CRP), a biomarker of vascular and systemic inflammation from a blood sample, was measured at Wave 7. Because CRP displayed a skewed distribution, it was transformed using log transformation to meet the assumption of linearity for inclusion in ordinary least squares regression.

### Analytic Strategy

All analyses were run using M*plus* 8.1. We used regression analyses to examine associations between harsh parenting in adolescence and change in BMI. We first checked for potential multicollinearity among variables. VIF scores ranged between 1.00 for harsh parenting and 1.06 for exercise, indicating no evidence of multicollinearity (VIF <10) among the study variables. Then, we used path modeling with maximum likelihood estimation to test our mediating hypothesized models. In the path models, we controlled for genetic risk score for obesity (GRSO) index and sociodemographic covariates. To assess goodness-of-fit of the model, we used Steiger's root-mean-square error of approximation (RMSEA < 0.05) and the comparative fit index (CFI > 0.90). The 95% confidence interval (CI) estimated with bias-corrected and accelerated bootstrapping with 1,000 resamples was used to assess the significance of hypothesized indirect effects (32). Finally, to address the robustness of our results, we tested for differences between the models for males and females using the multiple group analysis.

To test our hypotheses, variables were entered in the models in the following steps: (a) the main effect model, which tested the effects of harsh parenting (ages 10–15) on Δ BMI form age 10–29 (Hypothesis 1); (b) the indirect effect model with obesity-related obesity (ORDM) as a mediator, which was used to test an indirect effect of harsh parenting on Δ BMI through ORDM (Hypothesis 2); (c) the indirect effect model with ORDM and gene expression of obesity-related genes (GEOG) as mediators, which were used to test the pathway: harsh parenting → ORDM → GEOG → Δ BMI aging (Hypothesis 3).

## Results

### Initial Findings

Mean BMI was 21.776 (*SD* = 5.746) at Wave 1 (age 10) and 31.281 (*SD* = 8.181) at Wave 7 (age 29). At Wave 7 about 47% of participants had a BMI >30, which is considered obese. Correlations, means, and standard deviations for all study variables are displayed in Table 1. As expected, there were significant correlations of harsh parenting with change in BMI (Δ BMI) from ages 10–29 (r = 0.115, *p* = 0.029) and obesity-related DNAm (ORDM) (r = −0.185, *p* < 0.001). Further, ORDM was significantly related to gene expression of obesity-related genes (GEOG) (r = −0.178, *p* < 0.001) and Δ BMI (r = −0.374, *p* < 0.001). Finally, GEOG was significantly and positively associated with Δ BMI (r = 0.286, *p* < 0.001). Because all elements of potential mediation emerged, we proceeded with a formal test of our mediating hypotheses.

**Table 1:** Correlations, means, and standard deviations among study variables (*N* = 362).

	**1**	**2**	**3**	**4**	**5**	**6**	**7**	**10**		
1. Δ BMI (ages 10–29)	–									
2. Harsh parenting (ages 10–15)	0.115*	–								
3. Obesity-related DNAm	−0.374**	−0.185**	–							
4. GEOG	0.286**	0.055	−0.178**	–						
5. Genetic risk score for obesity	0.110*	0.024	−0.110*	0.062	–					
6. Males	−0.242**	−0.021	0.188**	−0.214**	−0.014	–				
7. Log-income	−0.048	−0.009	0.186**	−0.030	−0.038	0.044	–			
8. Exercise	−0.108*	0.031	0.080	−0.145**	0.047	0.186**	0.111*	–		
9. Unhealthy diet	−0.032	0.135**	−0.056	0.124*	−0.054	−0.069	−0.075	0.005	–	
10. Log-CRP	0.438**	0.086	−0.207**	0.273**	0.082	−0.229**	0.022	−0.065	0.000	–
Mean	0.000	1.608	0.366	3.874	0.276	0.360	7.988	2.593	3.123	0.868
*SD*	7.796	0.279	0.00	0.203	0.083	0.481	4.115	1.156	1.037	0.579

* *p ≤ 0.05*;

** *p ≤ 0.01 (two-tailed tests). GEOG, Gene expression of obesity-related genes; Δ = change in BMI from ages 10 (wave 1) to 29 (wave 7)*.

We first checked for evidence of gene-environment correlation (rGE). As presented in Table 1, there was no significant direct association of genotype with harsh parenting (r = 0.024, *ns*), suggesting that there was no evidence of an rGE whereby elevated genetic risk for obesity led to increased exposure to harsh parenting. In addition, gender, income, exercise, and genetic risk score for obesity showed association with either dependent variables or mediators. The results suggest the value of suggesting the value of retaining them as controls in the analyses.

### Hypothesis 1: Effect of Harsh Parenting on Change in BMI

Table 2 presents the effect of harsh parenting on Δ BMI. Consonant with the correlation matrix, harsh parenting in adolescence was significantly associated with Δ BMI (β = 0.115, *p* < 0.029). Next, as hypothesized, Model 2 shows that this association (β = 0.117, *p* < 0.022) was maintained after controlling for gender, income, exercise, and genetic risk score for obesity. Consistent with prior research (31), the beta coefficients for genetic risk score for obesity was positively related to Δ BMI, and being male was also significantly associated with Δ BMI (b = −3.695, *p* < 0.001).

**Table 2:** Regression models examining harsh parenting as a predictor of change in body mass index (*N* = 362).

	**Δ BMI (ages 10–29)**			
	**Model 1**		**Model 2**	
**Variables**	***b***	**β**	***b***	**β**
Harsh parenting (ages 10–15)	0.894*	0.115	0.915*	0.117
	(0.408)		(0.399)	
Genetic risk score for obesity			9.698*	0.103
			(4.786)	
Males			−3.695**	−0.228
			(0.837)	
Income			−0.051	−0.027
			(0.097)	
Exercise			−0.472	−0.070
			(0.352)	
Unhealthy diet			−0.445	−0.059
			(0.387)	
Constant	0.000		1.682	
	(0.408)		(2.207)	
*R*-square	0.013		0.091	

*Unstandardized (b) and standardized (β) coefficients shown, with standard errors in parentheses; harsh parenting (ages 10–15) is standardized by z-transformation (mean = 0 and SD =1); income is log transformed; Δ = change in BMI from ages 10 (wave 1) to 29 (wave 7)*.

* *p ≤ 0.05*;

** *p ≤ 0.01 (two-tailed tests)*.

### Hypothesis 2: Obesity-Related DNAm Mediates the Effect of Harsh Parenting on Change in BMI

**Figure 2:**
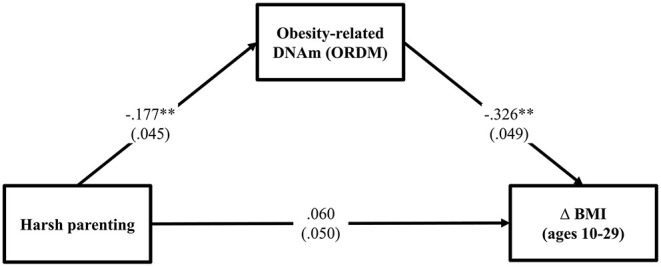
Methylation of obesity-related genes mediates the impact of harsh parenting on change in body mass index (*N* = 362). Chi-square = 0.512, *df* = 2, *p* = 0.774; CFI = 1.000; RMSEA = 0.000. Values are standardized parameter estimates and standard errors are in parentheses. Genetic risk score for obesity, males, income, exercise, unhealthy diet are controlled in these analyses. Δ = change in BMI from ages 10 (wave 1) to 29 (wave 7). **p ≤ 0.01; *p ≤ 0.05 (two-tailed tests).

To test the hypothesis that ORDM mediates the impact of harsh parenting on change in BMI, we examined mediation using path modeling. As can be seen in Figure 2, fit indices indicate that the model fits the data well (χ^2^ = 0.512, *df* = 2, *p* = 0.774; CFI = 1.000; RMSEA = 0.000). Controlling for gender, income, exercise, unhealthy diet, and genetic risk score for obesity, the model shows that harsh parenting in adolescence was related to ORDM (β = −0.177, *p* < 0.000), which in turn, was related to change in BMI across adolescence and young adulthood (β = −0.326, *p* < 0.001). In addition, the previously significant effect of harsh parenting on Δ BMI was no longer significant when ORDM was included in the model (β = 0.060, *ns*). To better explicate the relative strength of the direct and indirect effects from harsh parenting to Δ BMI, we used the approach outlined by Preacher et al. (32) to compute indirect effects. Using a bootstrapping method with 1,000 replications, corrected for non-normality and asymmetrical confidence intervals, we found that the indirect effect of harsh parenting in adolescence on change in BMI from ages 10–29 was significant (indirect effect = 0.058, 95% CI [0.025, 0.093]). To compute the proportion of the total effect accounted for by the mediator, we calculated the amount the direct effect was reduced due to the introduction of the mediator, and divided by the total effect (33). Therefore, controlling for sociodemographic covariates and genetic risk score for obesity previously found to be associated with BMI among African Americans, ORDM accounted for about 53.2% of the total effect in Δ BMI explained by harsh parenting in adolescence. The second hypothesis was supported.

### Hypothesis 3: The Effect of Harsh Parenting on Change in BMI Through Obesity-Related DNAm and Gene Expression of Obesity-Related Genes

**Figure 3:**
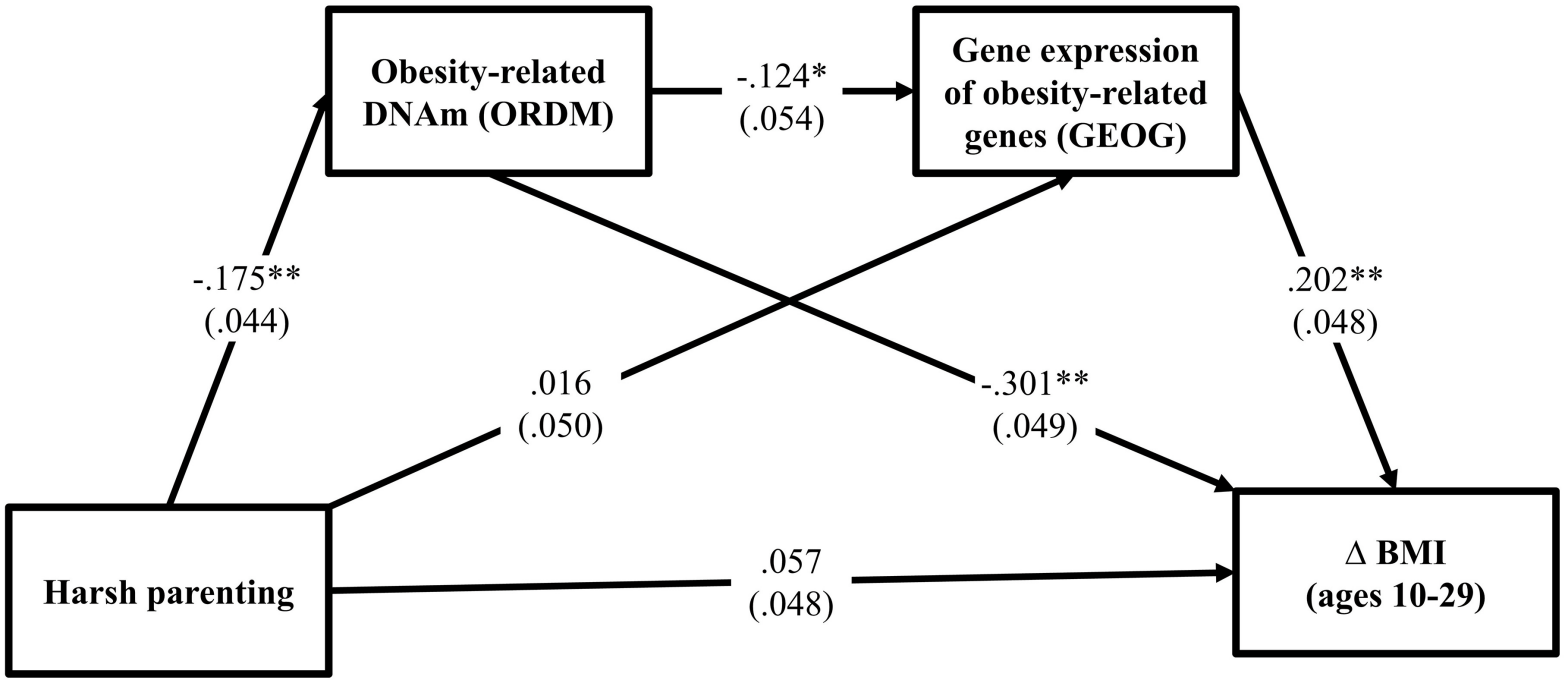
Methylation and gene expression of obesity-related genes mediates the impact of harsh parenting on change in body mass index (*N* = 362). Chi-square = 0.721, *df* = 2, *p* = 0.698; CFI = 1.000; RMSEA = 0.000. Values are standardized parameter estimates and standard errors are in parentheses. Genetic risk score for obesity, males, income, and unhealthy diet are controlled in these analyses. Δ = change in BMI from ages 10 (wave 1) to 29 (wave 7). **p ≤ 0.01; *p ≤ 0.05 (two-tailed tests).

Turning to the third hypothesis, GEOG was added into the path between ORDM and Δ BMI. As can be seen in Figure 3, the fit indexes were good for this model (χ^2^ = 0.721, *df* = 2, *p* = 0.698; CFI = 1.000; RMSEA = 0.000). As expected, harsh parenting in adolescence predicted demethylated ORDM (β = −0.175, *p* < 0.000) that in turn predicted both GEOG (β = −0.124, *p* = 0.021) and Δ BMI (β = −0.301, *p* < 0.001). Further, GEOG was significantly related to Δ BMI (β = 0.202, *p* < 0.001). Table 3 summarizes the results using the bootstrapping method with 1,000 replications to test the significance of direct and indirect effects. The table shows that two indirect pathways are significant. This includes the path harsh parenting → ORDM → GEOG → Δ BMI (indirect effect = 0.004, 95% CI [0.001, 0.012], with a small-medium effect size 0.034), as well as harsh parenting → ORDM → Δ BMI (indirect effect = 0.053, 95% CI [0.024, 0.089], with a large effect size 0.453). Overall, the results provide support for the hypothesized model, but do not indicate that all effects of obesity-related DNAm on BMI are mediated by the expression of the genes in the ORDM index.

**Table 3:** Summary of indirect effects.

**Paths**	**Indirect effect** **95% CI**	**% of variance** **for mediator**
Harsh parenting → ORDM → GEOG → Δ BMI	0.004 95% CI (0.001, 0.012)	3.42%
Harsh parenting → ORDM → Δ BMI	0.053 95% CI (0.024, 0.089)	45.29%

*Bootstrapping with 1,000 replications; 95% CI, 95% confidence interval; ORDM, Obesity-related DNAm; GEO, Gene expression of obesity-related genes Δ = change in BMI from ages 10 (wave 1) to 29 (wave 7)*.

### Sensitivity Analysis

To address the robustness of our findings, we tested for differences between the models for women and men using multiple group analyses. We began by estimating a model that constrained the paths for women and men to be equal. Next, we estimated a model that freed the paths to vary by gender. The chi-square difference between the models was significant, indicating structural non-invariance. That is, the model fit was significantly worse when the paths were constrained to be equal for men and women. To determine which paths were different, we freed one path in the constrained model at a time and compared it with the constrained model's chi-square with 1 degree of freedom. Table 4 shows that there was no gender difference in various paths [harsh parenting → ORDM: $\chi_{\left(1\right)}^{2}$ = 0.149, *p* = 0.699; ORDM → GEOG: $\chi_{\left(1\right)}^{2}$ = 3.336, *p* = 0.068; GEOG → Δ BMI: $\chi_{\left(1\right)}^{2}$ = 0.002, *p* = 0.964, respectively]. Finally, to test our assumption that ORDM led to GEOG and not vice versa, we tested this possibility directly. As shown in online Supplementary Figure 1, the alternative model, that GEOG led to ORDM was not supported because harsh parenting was not associated with gene expression of obesity-related genes (GEOG). Finally, given that healthy and unhealthy obesity may be differently involved in physiological processes (34), we repeated the analysis presented in Figure 3 controlling for C-reactive protein (CRP), a marker of chronic inflammation associated with obesity that may have effects on DNA methylation and gene expression values. As presented in Supplementary Figure 2, the pattern of results was identical to that depicted in Figure 3.

**Table 4:** Multiple group comparison between females and males.

**Paths**	**χ^2^**	***Df***	$\Delta \chi_{\left(1\right)}^{2}$	***p*** **-value**
Harsh parenting → ORDM				
*b*s equal for both	36.892	20		
*b*s free to differ	36.743	19	0.149	0.699
ORDM → GEOG				
*b*s equal for both	36.892	20		
*b*s free to differ	33.556	19	3.336	0.068
GEOG → Δ BMI				
*b*s equal for both	36.892	20		
*b*s free to differ	36.890	19	0.002	0.964

*ORDM, Obesity-related DNAm; GEOG, Gene expression of obesity-related genes*.

## Discussion

The current study examined a plausible mediational model linking harsh parenting in adolescence with BMI in young adulthood *via* changes in DMA methylation and gene expression for genes known to be related to BMI among African Americans. Whereas, most studies have been cross-sectional and have used retrospective reports of early adversity (35), we used longitudinal data to examine prospective associations between harsh parenting across childhood and adolescence to examine their association with change in BMI from childhood to young adulthood. Confirming prior retrospective research, prospectively reported adolescent harsh parenting was associated with the change in BMI from childhood to young adulthood, even after controlling for health-related covariates and genetic effects. It should be noted that our results indicated that an unhealthy diet shows no effect on obesity. Indeed, research on the association between nutrients and obesity/health has produced mixed results (18, 36), especially self-reported measures. This is due, in part, to known limitations of self-reported measures of diet such as forgetting, biases, and distortions common to all recall measures (7, 37). Accordingly, our findings are consistent with prior studies indicating that early stress experiences “get under the skin” and influence physiological processes through the life course (11, 12).

Previous studies have documented the effects of early childhood life stress on subsequent biological and genomic functioning (38), whereas our results suggest that harsh parenting during adolescence also exerts an influence on genomic functioning in young adults. To the best of our knowledge, this is the first study to examine whether obesity-related patterns of methylation might be responsive to family stress and help explain the link between harsh parenting experienced during adolescence and BMI in young adulthood. Given that the levels of DNA methylation vary depending on environmental influences, we posited that harsh parenting in adolescence would be associated with obesity-related DNAm; and that the level of obesity-related DNAm would be associated with the change in BMI from childhood to young adulthood. These predicted associations were all significant using the current sample of African Americans, net of the contribution of genetic variability, gender, income, and exercise. Hence methylation might be seen providing the biological underpinnings for linking between environments and phenotypes.

In addition, DNA methylation plays a significant role in regulating gene expression (20). An integrated model that incorporates social factors, DNA methylation, and gene expression provides a more nuanced understanding of the potential biosocial mechanisms that may link relevant social context to health outcomes. The present study shows that obesity-related DNAm mediates the effect of harsh parenting on increased gene expression of obesity-related genes which, in turn, partially accounts for the development of obesity. Therefore, one important finding from the current study was that methylation and gene expression emerged as plausible biological mediators, and strengthened the case for social policies and interventions to enhance family environments during childhood and adolescence as one component of a multi-pronged effort to address the obesity epidemic (39).

It is important to acknowledge that many different types of positive and negative childhood adversities and stressors have been implicated as potential precursors of obesity—and these factors have a strong tendency to co-occur (40–42). Moreover, the role of structural racism creates toxic ecological environments that undermine the health of Black Americans by limiting their access to various resources (43, 44) and exposing them to childrearing failure (45). Indeed, the social marginality and economically adverse that characterize many Black neighborhoods (46) has been shown to increase harsh parenting and harm various health problems through various social and biological pathways (47). Accordingly, it is possible that an assessment of cumulative stresses across childhood and youth, including adversities arising inside the family, within the neighborhood, within school settings, or within the broader society, might better predict adult obesity and health outcomes than does harsh parenting alone (40). Similarly, it is important to acknowledge complexity in the assessment of family environment for African American youth. Although some studies have suggested an increased likelihood of African Americans experiencing reported childhood maltreatment (48, 49). Acceptability of corporal punishment among African Americans may result in greater reports of “harsh parenting” that reflect some mixture of no nonsense parenting as well as exposure to abusive parenting (50). Poverty and stressful neighborhood characteristics are also associated with relatively harsher parenting strategies (51), increasing the likelihood of exposure to physical abuse among those raised in low-income communities (52). No nonsense parenting involves physical punishment and the use of physical restraint but occurs within the context of warmth and affection (53). It should also be noted that poverty and stressful neighborhood characteristics are associated with relatively harsher parenting strategies (54), and these factors may also contribute indirectly to the development of obesity. Accordingly, although reports of harsh parenting likely reflect higher stress environments, it cannot be assumed they reflect abusive parenting or even inappropriate parenting for some difficult contexts.

Limitations of the current study also should be noted. First, these findings, which are based on an African American sample and await replication with other ethnicities. It is possible that different social processes may emerge as central for other ethnic groups. Further, it should also be noted that the timing of methylation and gene expression assessments relative to the assessment of young adult BMI does not allow us to rule out causal effects from BMI to methylation and gene expression. Accordingly, future work with multiple assessments, preferably including assessment in late childhood as well as early adulthood, will be necessary to better examine direction of effects between methylation, gene expression, early harsh parenting practices, and change in BMI. Third, gene regulation may contribute to other aspects of health outcomes beyond obesity, suggesting the importance of testing for potential associations with other aspects of cardiometabolic health as youth reach the age of increased risk. This may help explicate why African American adults are twice as likely as European American adults to die of heart disease and diabetes (55) and how this disparity comes to be rooted in childhood experiences associated with economic stress and early deprivation (56). Finally, our results suggest a biosocial mechanism that links the early family environment to long-term obesity problems mediated by epigenetic pathways. However, our findings do not rule out potential additional mechanisms linking harsh parenting and obesity. Obesity is known to increase the chances of developing dyslipidemia, characterized by elevated plasma triglycerides, reduced high-density lipoprotein (HDL) cholesterol, and elevated apoB concentration (57). Further, the change of dyslipidemia also, in turn, affects DNA methylation and induces changes in gene expression profiles (58, 59), suggesting additional mechanistic pathways to examine. In particular, although BMI is strongly related to both subcutaneous and visceral adipose tissues (60), changes in these tissues may have different effects on metabolic disorders. Therefore, future research that can better distinguish changes in subcutaneous and visceral adipose tissues will be needed to better understand the association of harsh parenting to each.

Given that obesity is assoicated with earlier onset of cardiovascular disease (31), the current results suggest that one way in which harsh parenting in adolescence may influence long-term health outcomes such as obesity is by acting on gene regulation. Identifying the mechanisms linking early experiences with later health consequences can inform the development of new preventive interventions by identifying potential boundary conditions for program efficacy and providing enhanced measurement strategies for detecting positive change. From a social policy standpoint, the current results suggest that modifiable family influences may exert an effect on obesity. In turn, this suggests that programs to promote healthy family interaction can improve long-term health outcomes by reducing propensity toward obesity. Future research should examine whether some of the apparent impacts of epigenetic change on BMI may result from associations with cognitive and behavioral changes that act in concert with biological pathways to obesity. Explication of biological effects on earlier behavioral phenotypes (e.g., lifestyles) may enhance the forecasting of outcomes in young adulthood, leading to the identification of additional preventive intervention targets and clarifying relevant developmental processes conferring risk. In particular, research identifying relevant stress proliferation processes may be useful in expanding the current model. It will also be important to examine additional epigenetic mechanisms given the many genes potentially relevant to obesity.

## Data Availability Statement

The original contributions presented in the study are included in the article/Supplementary Materials, further inquiries can be directed to the corresponding author.

## Ethics Statement

The studies involving human participants were reviewed and approved by FACHS weathering-Targets, Study approval number 00006152. Written informed consent to participate in this study was provided by the participants' legal guardian/next of kin.

## Author Contributions

M-KL led design and analysis, participated in the construction of measures, and drafted the manuscript. SB participated in the study's design and drafting of the manuscript. RS and KY conceived of the study and made substantive contributions to the manuscript regarding the interpretation of findings. All authors contributed to the article and approved the submitted version.

## Funding

This research was supported by Award Number R01 HL8045 from the National Heart, Lung, and Blood Institute, R01 HD080749 from the National Institute of Child Health and Human Development, R01 AG055393 from the National Institute on Aging, and R01 CA220254 from the National Cancer Institute.

## Conflict of Interest

The authors declare that the research was conducted in the absence of any commercial or financial relationships that could be construed as a potential conflict of interest.

## Publisher's Note

All claims expressed in this article are solely those of the authors and do not necessarily represent those of their affiliated organizations, or those of the publisher, the editors and the reviewers. Any product that may be evaluated in this article, or claim that may be made by its manufacturer, is not guaranteed or endorsed by the publisher.
