# Supplementary material for: The Impact of Harsh Parenting on the Development of Obesity in Adulthood: An Examination of Epigenetic/Gene Expression Mediators Among African American Youth
Source: Front Cardiovasc Med. 2021 Nov 5;8:755458. doi: 10.3389/fcvm.2021.755458 (PMC8602565; doi:10.3389/fcvm.2021.755458)
Supplement: Supplementary file 1 [file Data_Sheet_1.docx]

frontier in cardiovascular medicine

ONLINE SUPPLEMENT

The impact of harsh parenting on the development of obesity in adulthood: An examination of epigenetic/gene expression mediators among African American youth

**Table S1.**

Comparisons of participants who did or did not have completed data for the current study

|  | With  Completed Data  (*n* = 362) | |  | Without Completed Data  (*n* = 527) | |  |  |
| --- | --- | --- | --- | --- | --- | --- | --- |
|  | M | *SD* |  | M | *SD* | *t*-value | *p-*value |
| Harsh parenting (Wave 1) | 1.597 | .336 |  | 1.575 | .333 | .937 | .349 |
| Body mass index (Wave 1) | 21.776 | 5.746 |  | 21.783 | 5.523 | -.017 | .986 |
| Family income (Wave 1) | 38828.976 | 35412.606 |  | 40036.784 | 36464.033 | -.491 | .624 |
| Parental education (Wave 1) | 13.378 | 2.269 |  | 13.547 | 2.418 | -1.049 | .294 |

**
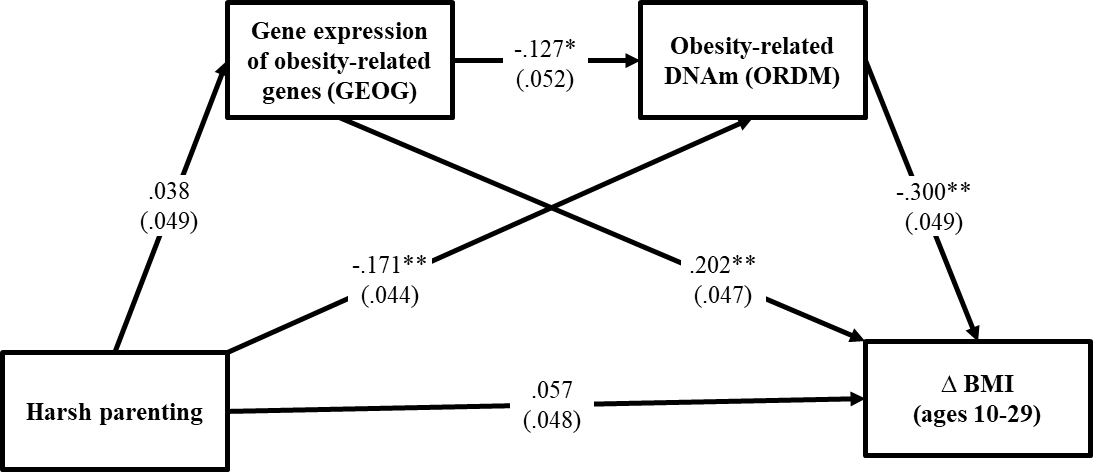
**

**Figure S1.** Gene expression of obesity-related genes and obesity-related DNAm mediates the impact of harsh parenting on change in body mass index (*N* = 362)

*Note*. Chi-square = .156, *df* = 2, *p* = .925; CFI = 1.000; RMSEA = .000. Values are standardized parameter estimates and standard errors are in parentheses. Genetic risk score for obesity, males, income, and exercise are controlled in these analyses. Δ = change in BMI from ages 10 (wave 1) to 29 (wave 7).

***p* ≤ .01; **p* ≤ .05 (two-tailed tests).

**
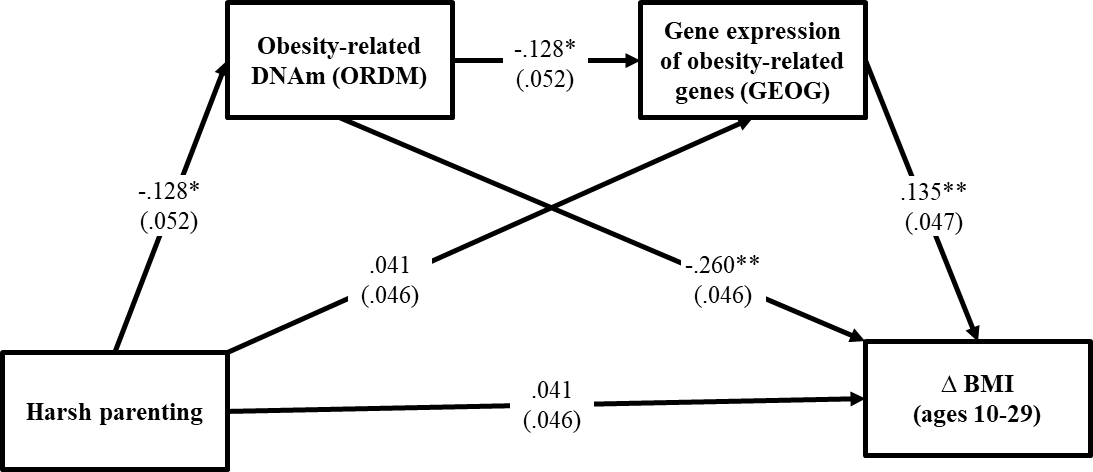
**

**Figure S2.** Gene expression of obesity-related genes and obesity-related DNAm mediates the impact of harsh parenting on change in body mass index after controlling for log C-reactive protein (*N* = 362)

*Note*. Chi-square = 25.826, *df* = 8, *p* = .001; CFI = .917; RMSEA = .078. Values are standardized parameter estimates and standard errors are in parentheses. Genetic risk score for obesity, males, income, and exercise are controlled in these analyses. Δ = change in BMI from ages 10 (wave 1) to 29 (wave 7).

***p* ≤ .01; **p* ≤ .05 (two-tailed tests).
